# Paroxysmal extreme pain disorder M1627K mutation in human Na_v_1.7 renders DRG neurons hyperexcitable

**DOI:** 10.1186/1744-8069-4-37

**Published:** 2008-09-19

**Authors:** Sulayman D Dib-Hajj, Mark Estacion, Brian W Jarecki, Lynda Tyrrell, Tanya Z Fischer, Mark Lawden, Theodore R Cummins, Stephen G Waxman

**Affiliations:** 1 Deptartment of Neurology, Yale University School of Medicine, New Haven, CT 06510, USA; 2 Center for Neuroscience & Regeneration Research, Yale University School of Medicine, New Haven, CT 06510, USA; 3 Rehabilitation Research Center, VA Connecticut Healthcare System, West Haven, CT 06516, USA; 4 Dept. of Pharmacology & Toxicology, Stark Neurosciences Institute, Indiana University School of Medicine, Indianapolis, IN 46202, USA; 5 Neurology Department, Leicester General Hospital, Leicester, LE5 4PY, UK

## Abstract

**Background:**

Paroxysmal extreme pain disorder (PEPD) is an autosomal dominant painful neuropathy with many, but not all, cases linked to gain-of-function mutations in *SCN9A *which encodes voltage-gated sodium channel Na_v_1.7. Severe pain episodes and skin flushing start in infancy and are induced by perianal probing or bowl movement, and pain progresses to ocular and mandibular areas with age. Carbamazepine has been effective in relieving symptoms, while other drugs including other anti-epileptics are less effective.

**Results:**

Sequencing of *SCN9A *coding exons from an English patient, diagnosed with PEPD, has identified a methionine 1627 to lysine (M1627K) substitution in the linker joining segments S4 and S5 in domain IV. We confirm that M1627K depolarizes the voltage-dependence of fast-inactivation without substantially altering activation or slow-inactivation, and inactivates from the open state with slower kinetics. We show here that M1627K does not alter development of closed-state inactivation, and that M1627K channels recover from fast-inactivation faster than wild type channels, and produce larger currents in response to a slow ramp stimulus. Using current-clamp recordings, we also show that the M1627K mutant channel reduces the threshold for single action potentials in DRG neurons and increases the number of action potentials in response to graded stimuli.

**Conclusion:**

M1627K mutation was previously identified in a sporadic case of PEPD from France, and we now report it in an English family. We confirm the initial characterization of mutant M1627K effect on fast-inactivation of Na_v_1.7 and extend the analysis to other gating properties of the channel. We also show that M1627K mutant channels render DRG neurons hyperexcitable. Our new data provide a link between altered channel biophysics and pain in PEPD patients.

## Background

Recent genetic studies have identified sodium channel Na_v_1.7 as a major contributor to pain [1,2]. Na_v_1.7 is predominantly expressed in dorsal root ganglion (DRG) and sympathetic ganglion neurons [3-6], and specifically in most functionally-identified DRG nociceptive neurons [7]. Global knock-out of Na_v_1.7 in mice is neonatal lethal while nociceptor-specific knock-out results in elevated mechanical and thermal pain thresholds and an attenuated pain response [8]. Surprisingly, congenital loss of Na_v_1.7 in humans is not associated with cognitive or motor deficits, but causes complete indifference to pain [9-11]. In contrast, one set of gain-of-function mutations of Na_v_1.7 in inherited erythromelalgia (IEM) leads to severe episodes of pain, mostly in the feet and hands [12-18], and a different set of mutations results in paroxysmal extreme pain disorder (PEPD) [19]. Because some cases of PEPD do not carry mutations in Na_v_1.7 [19], it is important to genetically profile new PEPD cases to determine the molecular basis of the disease which may influence possible therapy options.

Severe pain in PEPD patients accompanied by redness in the lower body can start in infancy, is induced by bowel movement or probing of perianal areas, and can be accompanied by tonic nonepileptic seizures, syncopes, bradycardia and occasionally asystole [20]. The cause for the seizures and cardiac symptoms is not well understood. Pain progresses with age to affect ocular and maxillary/mandibular areas and is triggered by cold, eating or emotional state [20]. Pain episodes can last seconds to minutes (and hours in extreme cases), and gradually subside.

Whole-cell voltage-clamp studies have shown that all of the IEM mutations lower the voltage threshold for Na_v_1.7 activation, with most mutations increasing the ramp response of the channel [13,16,17,21-25]. In contrast, PEPD mutations in Na_v_1.7 impair fast-inactivation and can result in a persistent current [19]. The M1627K mutation, which is located in domain IV S4–S5 linker (DIV/S4–S5) and first identified in a sporadic case of PEPD from France, has been shown to cause a significant shift in the voltage-dependence of steady-state fast-inactivation of mutant channels but does not produce a persistent current [19]. The effects of M1627K on other properties that influence channel function, for example slow-inactivation, recovery from fast-inactivation, and responses to ramp stimuli, have not been reported. Also, while it is predicted that impaired fast-inactivation could be linked to repetitive firing in hyperexcitable DRG neurons [26], the effects of the PEPD mutation on DRG neuron excitability have not been experimentally demonstrated.

We report here the identification of M1627K mutation in a new family with PEPD. We confirm that the M1627K mutation causes a large depolarized shift in fast-inactivation without altering channel activation, and show that mutant channels inactivate from the open state with slower kinetics, but without altering the rate for development of closed-state inactivation. We demonstrate that M1627K channels recover from fast-inactivation faster than wild type (WT) channels, and produce larger currents in response to a ramp stimulus. We also show, for the first time, that a PEPD mutation reduces the threshold for single action potentials and increases the number of action potentials in DRG neurons in response to graded stimuli, providing a link between altered channel biophysics and the pain that occurs in PEPD.

## Results

### Clinical phenotype

An English family with a history of PEPD in two generations was evaluated for a linkage with mutations in *SCN9A*. The proband is a 36 year old Caucasian female who presented to her physician with a lifelong history of episodic erythema and painful burning sensations from the waist downward. The episodes of erythema and extreme pain last for approximately 20 minutes. Her symptoms began in infancy and occurred several times a year, with pain triggered by bowel movement, passage of flatus or any painful stimulus in the lower half of her body. The patient experienced two attacks during each of her two deliveries, each of which occurred by Caesarian section under general anesthesia. Treatment with carbamazepine was initiated in infancy and currently has been successful in reducing her attacks to approximately one per year. As a teenager, the patient tried to reduce the daily dose; however, at 400 mg of carbamazepine per day she experienced an increase in the frequency of her attacks. Her symptoms dramatically improved upon returning to the original dose of 600 mg per day. Based on the clinical evaluation, the patient was diagnosed with PEPD.

The proband's father and sister have a similar presentation of the disease since infancy. The father is currently not treated with any medication and feels that the frequency of attacks has increased with age. The proband's sister has been treated effectively with carbamazepine since childhood, but requires a lower dose of 200 mg/day. Prior to starting carbamazepine at one year of age, she had frequent episodes of pain and erythema from the waist downward. She self-reports that her symptoms have improved with age, and currently she has suffered just two attacks in the last five years. Unlike the proband, she can recall three attacks in her arms triggered by minor hand injuries. She also had two attacks during Caesarian section under general anesthesia.

### Identification of M1627K mutation

**Figure 1:**
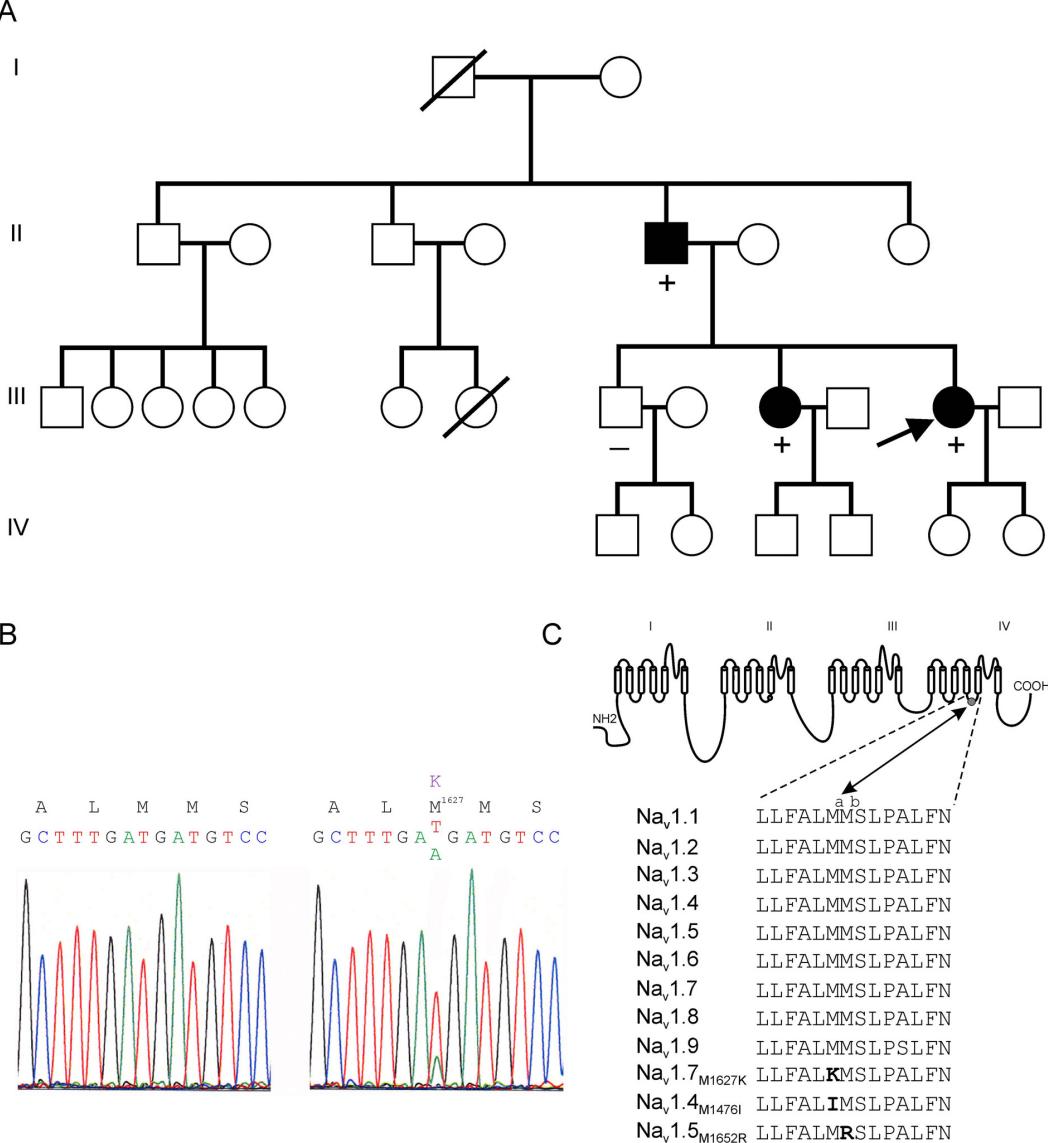
**Inheritance pattern of the M1627K mutation in DIV/S4–5 linker in Na_v_1.7 in familial PEPD**. ***A***, Inheritance of PEPD in two generations of a family with PEPD from the UK. Circles denote females; squares denote males, and symbols with diagonal line denote deceased individuals. The proband is indicated by an arrow. Blackened symbols indicate subjects affected with PEPD. ***B***, Sequence traces of a segment of exon 26 in the region which encodes M1627. Left trace shows homozygous T4879 from exon 26 of the unaffected brother of the proband. Right trace shows heterozygous T4879A in exon 26 from the proband. This missense mutation leads to M1627K substitution. In (***A***), a (+) symbol denotes subjects heterozygous for the T4879A mutation in exon 26, and a (-) symbol denotes subjects without the mutation. Inheritance of PEPD segregates with the T4879A heterozygosity. ***C***, Sequence alignment of DIV/S4–S5 linker from human sodium channels. The M1627K substitution in this family with PEPD is noted in the sequence from Na_v_1.7. The M1476I from a family with cold-induced myotonia occurs at the corresponding site as M1627 in Na_v_1.4. The M1652R from a family with LQT-3 occurs at the adjacent methionine residue in Na_v_1.5 (M1628 in Na_v_1.7).

Genomic DNA from the proband and her family (Figure 1A) was used to amplify all known exons of *SCN9A*, and their sequences were compared to the reference Na_v_1.7 cDNA [27]. Proband and control templates produced similar amplicons which were purified and sequenced. Sequence analysis identified a T to A in exon 26 (Figure 1B), corresponding to position 4879 of the reference sequence [27]. This mutation substitutes methionine (M) by lysine (K) at position 1627 of the polypeptide, which is located in the middle of the DIV/S4–5 linker. The missense mutation segregates with the disease in this family, with the affected father and a sister heterozygotes for the mutation and an unaffected brother carrying only the wild-type alleles (Figure 1A). M1627 is conserved in all human sodium channels (Figure 1C), and was previously identified independently in a sporadic case of PEPD from France [19].

### Voltage-clamp electrophysiology: activation and deactivation

**Figure 2:**
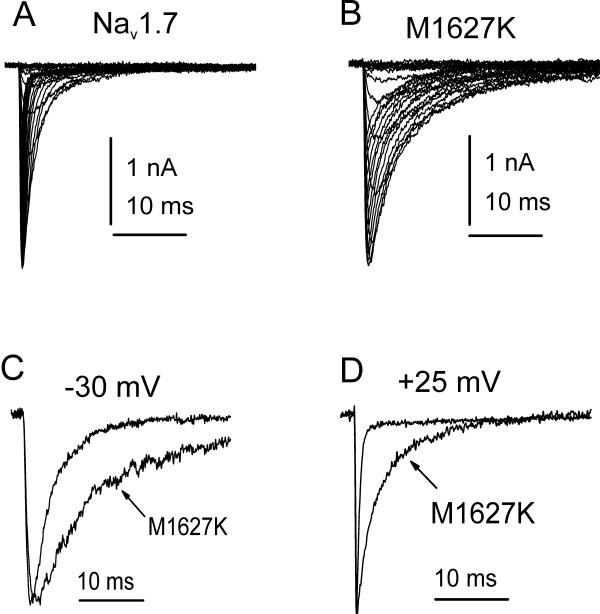
**M1627K currents decay slower than WT Na_v_1.7 currents**. Representative WT (***A***) and M1627K (***B***) Na_v_1.7 currents are shown. Cells were held at -100 mV and currents were elicited with 50 ms test pulses to potentials ranging from -80 to 40 mV. For better comparison, WT and M1627K currents elicited with -30 mV (***C***) and +25 mV (***D***) depolarizations are shown superimposed. Although the rate of activation is not apparently altered, the decay phase is substantially faster for WT currents.

**Figure 3:**
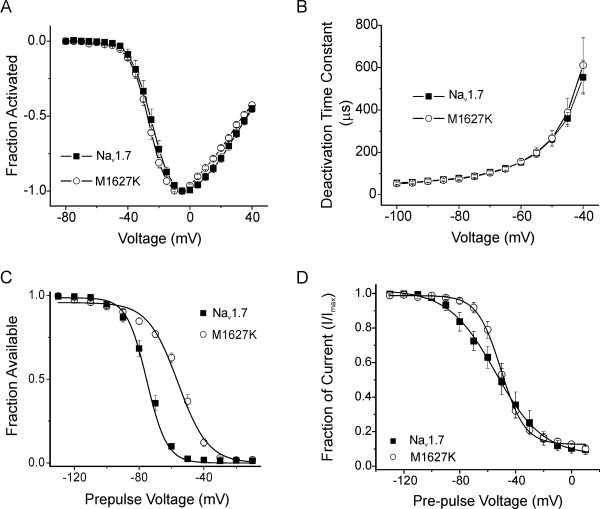
**The M1627K mutation alters inactivation properties of Na_v_1.7**. ***A***, Normalized peak current-voltage relationship for WT (filled squares, n = 11) and M1627K (open circles, n = 15) channels. Cells were held at -100 mV and currents were elicited with 50 ms test pulses to potentials ranging from -80 to 40 mV. ***B***, Time constants for tail current deactivation at repolarization potentials ranging from -40 to -100 mV for WT (filled squares, n = 8) and M1627K (open circles, n = 9) Na_v_1.7 channels. Time constants were obtained with single exponential fits to the deactivation phase of the currents. ***C***, Comparison of steady-state fast-inactivation for WT (filled squares, n = 11) and M1627K (open circles, n = 15) Na_v_1.7 channels. Currents were elicited with test pulses to 0 mV following 500 ms inactivating prepulses. ***D***, Comparison of slow-inactivation for WT (filled squares, n = 3) and M1627K (open circles, n = 4) Na_v_1.7 channels. Slow inactivation was induced with 10 s prepulses, followed by 100 ms pulses to -120 mV to allow recovery from fast-inactivation. A test pulse to 0 mV for 20 ms was used to determine the fraction of current available.

Sodium currents from WT hNa_v_1.7_R_, and the mutant channel M1627K were recorded from stably transfected HEK 293 cells in order to study the effects of this mutation on gating properties of the channel. Figure 2 shows representative WT (Figure 2A) and M1627K (Figure 2B) whole-cell currents elicited with a series of depolarizing test pulses from a holding potential of -100 mV. Although the peak current amplitude was smaller for M1627K channels (1.3 ± 0.2 nA, n = 15) than for WT channels (4.2 ± 0.7 nA, n = 12), the peak current-voltage relationship was similar for WT and M1627K channels (Figure 3A). The midpoint of activation (estimated by fitting the data with a Boltzman function) was -25.4 ± 1.0 mV (n = 14) for M1627K currents and -22.5 ± 1.9 mV (n = 11) for WT currents (p > 0.05). The kinetics of deactivation, which reflect the transition from the open to the closed state, of WT and M1627K channels were also examined by eliciting tail currents at a range of potentials after briefly activating the channels (at -20 mV for 0.5 ms). The time constants for deactivation were not altered at potentials ranging from -100 mV to -40 mV for the M1627K channel (Figure 3B). Together these data confirm that the M1627K mutation does not alter Na_v_1.7 activation properties.

### Voltage-clamp electrophysiology: inactivation

We next examined the effects of the M1627K mutation on inactivation properties. The voltage-dependence of steady-state fast-inactivation was dramatically shifted in the depolarizing direction by the M1627K mutation (Figure 3C). The midpoint of fast-inactivation (V_1/2_, measured with 500 ms pre-pulses) was -75.3 ± 1.7 mV for WT (n = 11) and -56.0 ± 1.4 mV for M1627K (n = 15) channels. The slope of the steady-state inactivation relationship was 6.2 ± 0.1 mV/e-fold for WT channels and 9.3 ± 0.6 mV/e-fold for M1627K channels. Differences in the V_1/2 _and slope of fast-inactivation fit were significant (p < 0.001). However, the fraction of current remaining available after the -10 mV inactivation prepulse was not significantly different (p > 0.05) between WT (1.3 ± 0.3%) and M1627K (1.9 ± 0.5%) channels.

By contrast, the voltage-dependence of slow-inactivation of hNa_v_1.7_R _currents was only slightly altered by the M1627K mutation (Figure 3D). Ten second pre-pulses, followed by 100 ms recovery pulses to -120 mV to allow recovery from fast-inactivation, preceded the test pulse (to 0 mV for 20 ms) to determine the fraction of current available. The M1627K mutation reduced the fraction of slow-inactivated mutant channels that occurred at -80, -70 and -60 mV, which might contribute to increased channel availability at normal resting membrane potentials.

**Figure 4:**
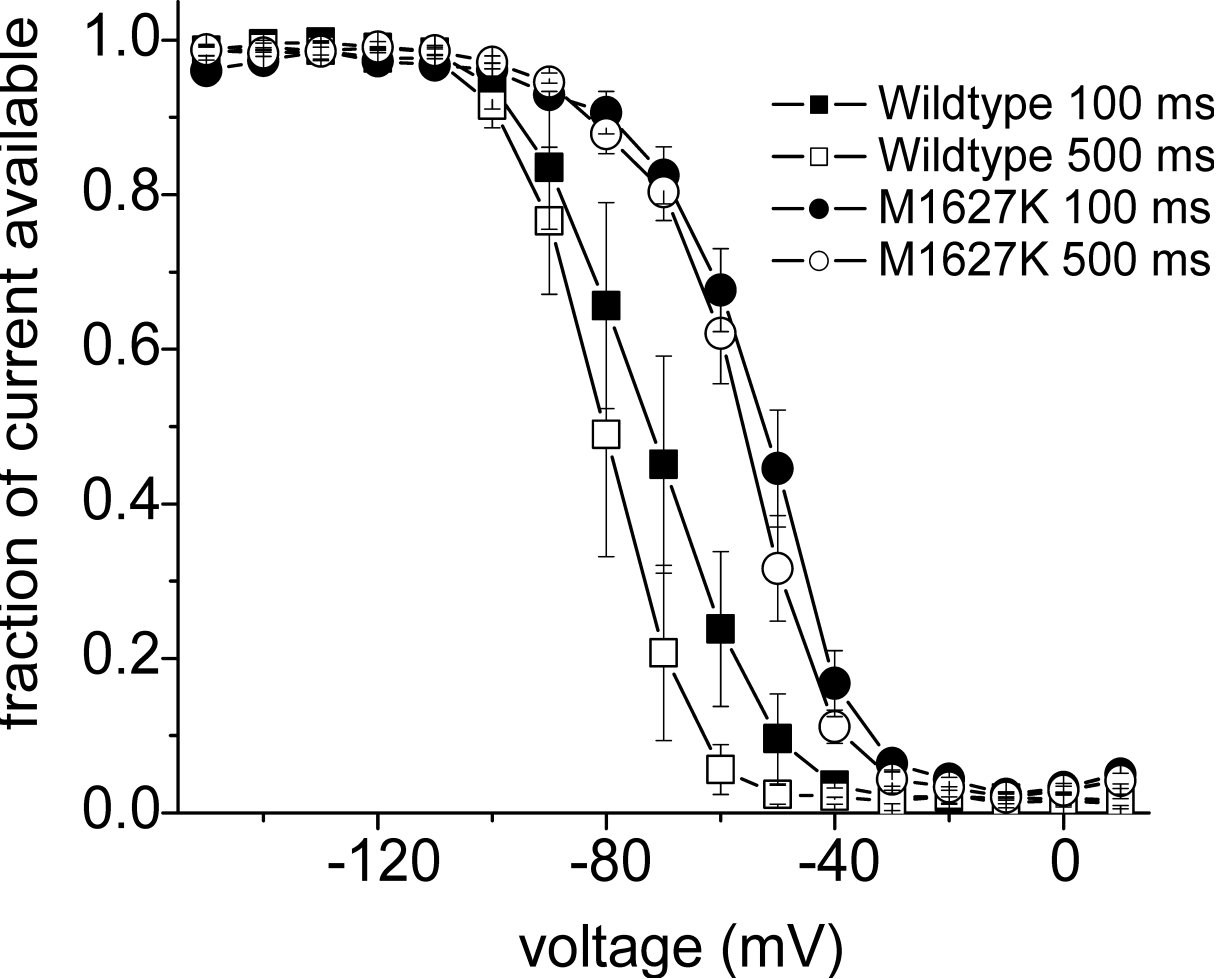
**Effect of prepulse duration on voltage-dependence of inactivation**. ***A***, Comparison of steady-state fast-inactivation for WT (squares, n = 3) and M1627K (circles, n = 4) Na_v_1.7 channels. Currents were elicited with test pulses to 0 mV following either 100 ms (filled symbols) or 500 ms (unfilled symbols) inactivating prepulses. The apparent voltage-dependence of inactivation of WT Na_v_1.7 channels is altered by the prepulse duration. In contrast the apparent voltage-dependence of inactivation of M1627K mutant channels is not significantly altered by changing the prepulse duration from 100 to 500 ms. Data are presented as mean ± S.E.

It should be noted that in this study we used 500 ms inactivating prepulses to determine the voltage-dependence of steady-state fast-inactivation. The time-constant for development of fast-inactivation at -70 mV is ~140 ms for WT channels [28] and therefore a 500 ms prepulse, which allows ~97% of the channels to inactivate at -70 mV, is ideal for measuring the voltage-dependence of steady-state fast-inactivation. One concern is that 500 ms could allow channels to also enter a state of slow-inactivation. Our data does not support this conclusion because 1) the time constant for development of slow-inactivation of WT channels at -50 mV was greater than 1000 ms and 2) accumulation of channels in a slow-inactivated state after 500 ms at -50 mV was less than 10% [29]. To further investigate this issue, we compared the voltage-dependence of WT and M1672K channel fast-inactivation using 100 ms prepulses (Figure 4). Although the inactivation curve for WT channels was more depolarized with the 100 ms prepulse than the 500 ms prepulse (due to incomplete fast-inactivation at negative potentials) the inactivation curves for M1672K channels were nearly identical (Figure 4). These data support the conclusion that the M1672K mutation causes a depolarizing shift in the voltage-dependence of fast-inactivation.

**Figure 5:**
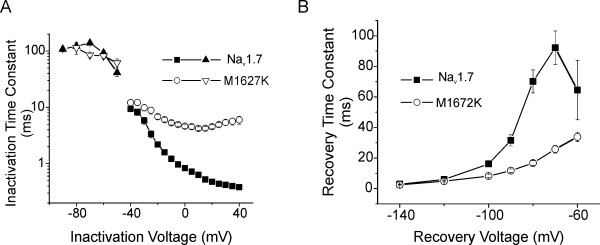
**The M1627K mutation alters rate of open-state inactivation and recovery from inactivation properties of Na_v_1.7**. ***A***, Time constants for development of fast-inactivation as a function of voltage for WT (filled symbols, n = 6) and M1627K (open symbols, n = 6) Na_v_1.7 channels are shown. Open-channel inactivation time constants were measured at voltages ranging from -45 to +40 mV by fitting the decay phase of currents elicited with depolarizing pulses with single exponentials. The time constants for development of closed-state inactivation were estimated from single exponential fits to time courses measured at inactivation potentials ranging from -90 to -50 mV for WT (filled triangles, n = 6) and with M1627K (open triangles, n = 6) Na_v_1.7 channels. ***B***, Recovery from inactivation kinetics are faster for M1627K mutant channels (open circles, n = 7) than for WT Na_v_1.7 channels (filled squares, n = 7). Time constants were estimated from single exponential fits to time courses measured at recovery potentials ranging from -140 to -60 mV. The recovery from inactivation voltage protocol involved prepulsing the cell to -20 mV for 20 ms to inactivate all of the current, then stepping the membrane potential back to the recovery potential for increasing recovery durations prior to the test pulse to 0 mV. The maximum pulse rate was 0.5 Hz.

Mutant channels exhibited slower decay kinetics (Figure 2) compared to WT channels, and we observed significant differences in the time constants for current inactivation between WT and M1627K channels. The rate of open-state inactivation was quantified by fitting the decay phase of the macroscopic current with a single exponential function. The time constants estimated from these fits are plotted as a function of the test potentials (Figure 5A). The time constants were much larger for M1627K currents than for WT currents over voltages that range from -30 to +40 mV. At -30 mV, for example, WT currents inactivated with a time constant of 5.8 ± 0.8 ms (n = 6) and M1627K currents inactivated with a time constant of 9.9 ± 1.3 ms (n = 6). At +25 mV, WT currents inactivated with a time constant of 0.44 ± 0.01 ms (n = 6) and M1627K currents inactivated with a time constant ten times larger (4.9 ± 0.4 ms, n = 6). These differences were statistically significant (p < 0.05). We also examined the development of closed-state inactivation at voltages ranging from -90 to -50 mV (Figure 5A, triangular symbols). Cells were stepped to the inactivation potential (from a holding potential of -100 mV) for increasing durations, and then stepped to the test potential (0 mV) to measure the fraction of the remaining available channels. The data from these cells were fitted with a single exponential function. Interestingly, the time course for the development of inactivation from the closed state was not significantly altered by the M1627K mutation, indicating that the M1627K mutation has a greater effect on open-state inactivation than closed-state inactivation.

### Voltage-clamp electrophysiology: recovery from fast-inactivation

The time course for recovery from fast-inactivation (repriming) of WT and M1627K channels was measured at recovery voltages ranging from -140 to -60 mV. Fast-inactivation was induced with 20 ms inactivating prepulses to -20 mV. The time course for recovery from inactivation for both WT and M1627K currents could be fitted with single exponential functions. Recovery from inactivation was significantly faster for M1627K channels than for WT channels (Figure 5B). For example, the time constant for recovery of WT channels (τ = 92 ± 11 ms, n = 7) was almost 4-fold larger at -70 mV than the corresponding time constant for M1627K channels (τ = 26 ± 3 ms, n = 7).

### Voltage-clamp electrophysiology: response to a ramp stimulus

**Figure 6:**
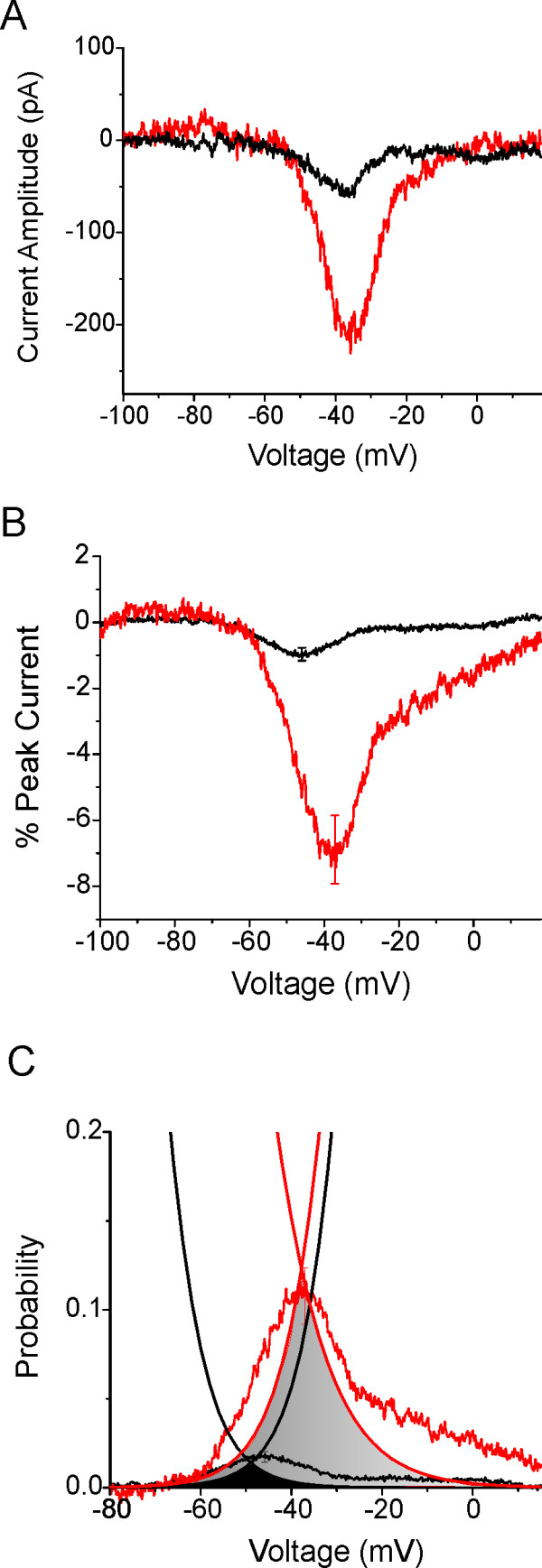
**The M1627K mutation increases the amplitude of currents elicited by slow ramp depolarizations**. ***A***, Representative ramp currents elicited by 600 ms long ramp depolarizations from -100 to +20 mV are shown recorded from a HEK293 cell expressing WT channels (black trace) and from one expressing M1627K channels (red trace). The peak transient current amplitude elicited in the WT cell was larger (3.4 nA) than that of the M1627K cell (2.8 nA). ***B***, The average relative ramp current (ramp current divided by peak transient current amplitude) is larger for M1627K cells (red trace, n = 9) than for WT cells (black trace, n = 7). ***C***, The properties of the averaged ramp currents are compared to the overlap between the voltage-dependence of activation (derived from the current-voltage relationship) and steady-state fast inactivation. The inverted amplitude of the WT ramp current was scaled so that it corresponded to the peak overlap between WT activation and steady-state fast inactivation (black shaded area). As can be seen, voltage-dependence of the WT ramp current corresponds to the region of overlap. Furthermore, the relative amplitude and voltage-dependence of the M1627K ramp currents corresponds to the overlap between M1627K activation and steady-state fast inactivation (red shaded area).

IEM mutations are known to produce enhanced responses to small, slow depolarizations (ramp stimuli) [1], but the ramp response has not been studied in PEPD mutations [19]. Figure 6A shows the ramp current recorded from a M1627K cell producing a current with peak amplitude of 2.8 nA, compared to the ramp current recorded from a WT cell producing a comparable current with a peak amplitude of 3.4 nA. The ramp currents elicited with slow ramp (0.2 mV/ms) depolarizations from -100 to +20 mV were expressed as a percentage of peak current (Figure 6B) and the relative amplitude of the ramp currents were significantly larger for M1627K channels (7.0 ± 1.0%; n = 9) than for WT channels (1.0 ± 0.2%; n = 7). We compared the relative amplitude of the ramp currents with the extent of overlap between the voltage-dependence of activation and steady-state fast-inactivation (Figure 6C). As shown, the relative amplitude of the WT and M1627K ramp currents, and the voltage-dependence of these currents, correlates reasonably well with the respective overlap between the voltage-dependence of activation and steady-state fast-inactivation.

### Current Clamp electrophysiology

Previous studies have not examined the effect of PEPD mutations on DRG neuron excitability [19]. We assessed the effect of the M1627K mutation on DRG neuron excitability by recording in current-clamp mode from DRG neurons transfected with GFP and either WT or the M1627K mutant construct. The GFP-positive cells that were selected for recording were similar for the two groups in cell size, as measured either by apparent cell body diameter (24.1 ± 0.6 μm for M1627K vs. 25.5 ± 0.5 μm for WT) or by measured whole-cell capacitance (23.4 ± 1.5 pF for M1627K vs. 23.6 ± 1.4 pF for WT). The expression of the M1627K mutant channels caused a significant reduction in the threshold to generate action potentials. DRG neurons (n = 28) that express WT channels required on average about 300 pA to reach threshold (292 ± 37 pA), whereas DRG neurons that express the M1627K mutant channel (n = 31) only required, on average, about half the amount of current to reach threshold (154 ± 20 pA; p < 0.005). One possibility for the reduction in threshold is an increase in input resistance that would give a larger depolarization to a fixed stimulus current. However, the difference of the input resistance between the two groups of transfected cells did not reach statistical significance (M1627K: 795 ± 105 MΩ; WT: 662 ± 67 MΩ; p = 0.3). A similar small, but non-significant, difference had been reported for the A863P IEM mutant [17].

No significant difference in resting membrane potential (RMP) was observed between the two groups of cells (M1627K: -60.1 ± 1.2 mV; WT: -60.1 ± 1.6 mV; p = 0.97). Additionally, the amplitude of action potential elicited at threshold showed no significant differences between M1627K transfected DRG neurons (n = 31) compared to WT transfected DRG neurons (n = 28) for peak (M1627K: 60.3 ± 2.2 mV, WT: 54.8 ± 2.9 mV, Mann-Whitney rank sum test, p = 0.24) or for after-hyperpolarization (M1627K: -64.2 ± 1.3 mV, WT: -59.2 ± 1.8 mV, t-test, p = 0.07).

**Figure 7:**
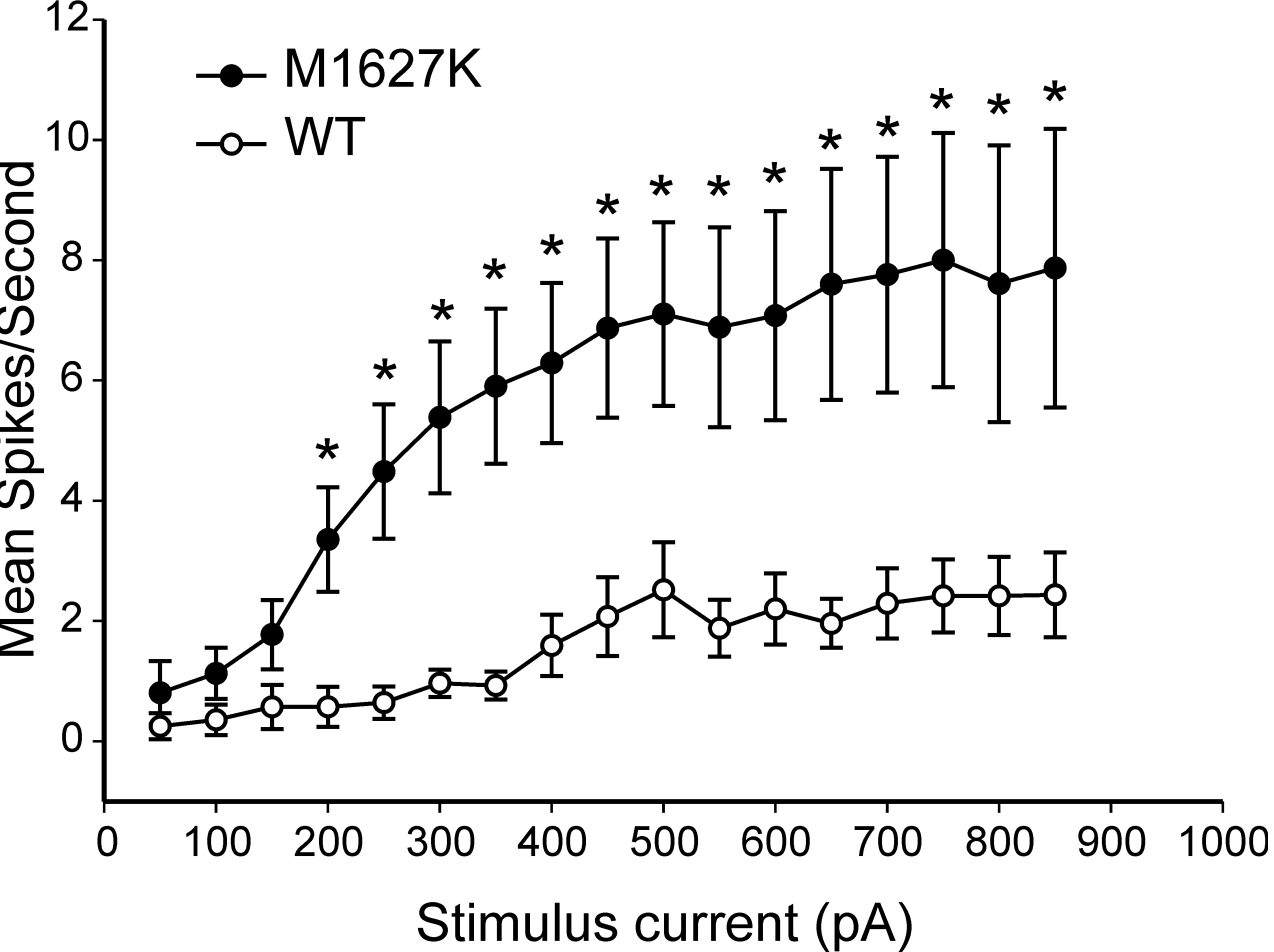
**M1627K mutant channels increase frequency of firing of DRG neurons compared to WT Na_v_1.7 channels**. The mean total number of action potentials (defined as spikes in membrane potential greater than 0 mV) for a current injection pulse of 1 second duration is plotted as a function of stimulus current intensity. The responses of neurons expressing M1627K mutant channels (n = 31) was significantly elevated over the responses of neurons expressing WT Nav1.7r channels (n = 28) for stimulus current injections exceeding 200 pA (p < 0.05).

**Figure 8:**
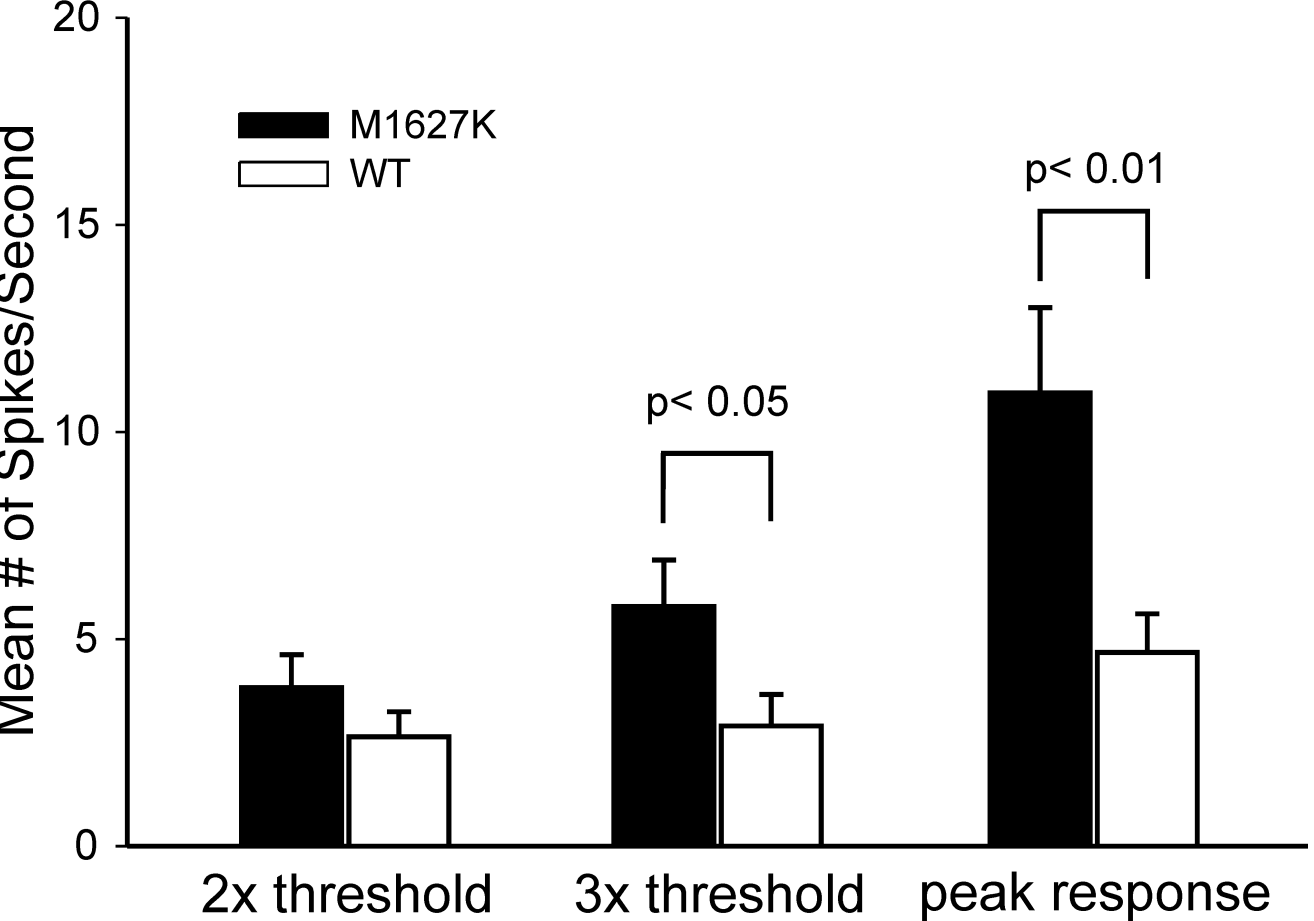
**Firing frequency is increased in current clamped DRG neurons expressing M1627K mutant channels when stimulus intensities are compared at fold-threshold**. For each cell the firing frequency at 2× and 3× threshold was determined and then averaged. Since threshold is lower for M1627K expressing neurons (154 ± 19 pA) compared to hNav1.7r-WT expressing neurons (292 ± 37 pA), the mean stimulus intensity at 2× and 3× was similarly scaled (319 ± 40 pA for M1627K versus 617 ± 68 for WT at 2× threshold; 483 ± 59 pA for M1627K versus 926 ± 101 pA for WT at 3× threshold). The firing frequency was significantly different between M1627K and WT for 3× threshold and peak firing frequency (p < 0.05 and p < 0.01) The lowest stimulus intensity that elicited the peak firing frequency was not significantly different (545 ± 50 pA for M1627K versus 564 ± 60 pA for WT).

DRG neurons that express the M1627K mutant channels fired more action potentials in response to suprathreshold graded stimuli compared to neurons that express WT channels. When the number of spikes elicited by a series of 1-second long depolarizing current injections was quantified, it is clear that on average the M1627K transfected cells responded with more spikes over the entire current injection range compared to WT transfected cells (Figure 7). The difference in firing was significantly different (p < 0.05) for the current injections between 200 pA to 750 pA. Since the threshold value for the M1627K mutant was significantly lower than for WT channel, one possible explanation would be that we are not comparing cells at the equivalent rheobase. Therefore, we carried out further analysis to compare these two groups as a function of fold-threshold. This analysis clearly demonstrated that the M1627K mutant causes increased excitability, reaching significance for 3× threshold as well for the maximum response that could be evoked (Figure 8).

**Figure 9:**
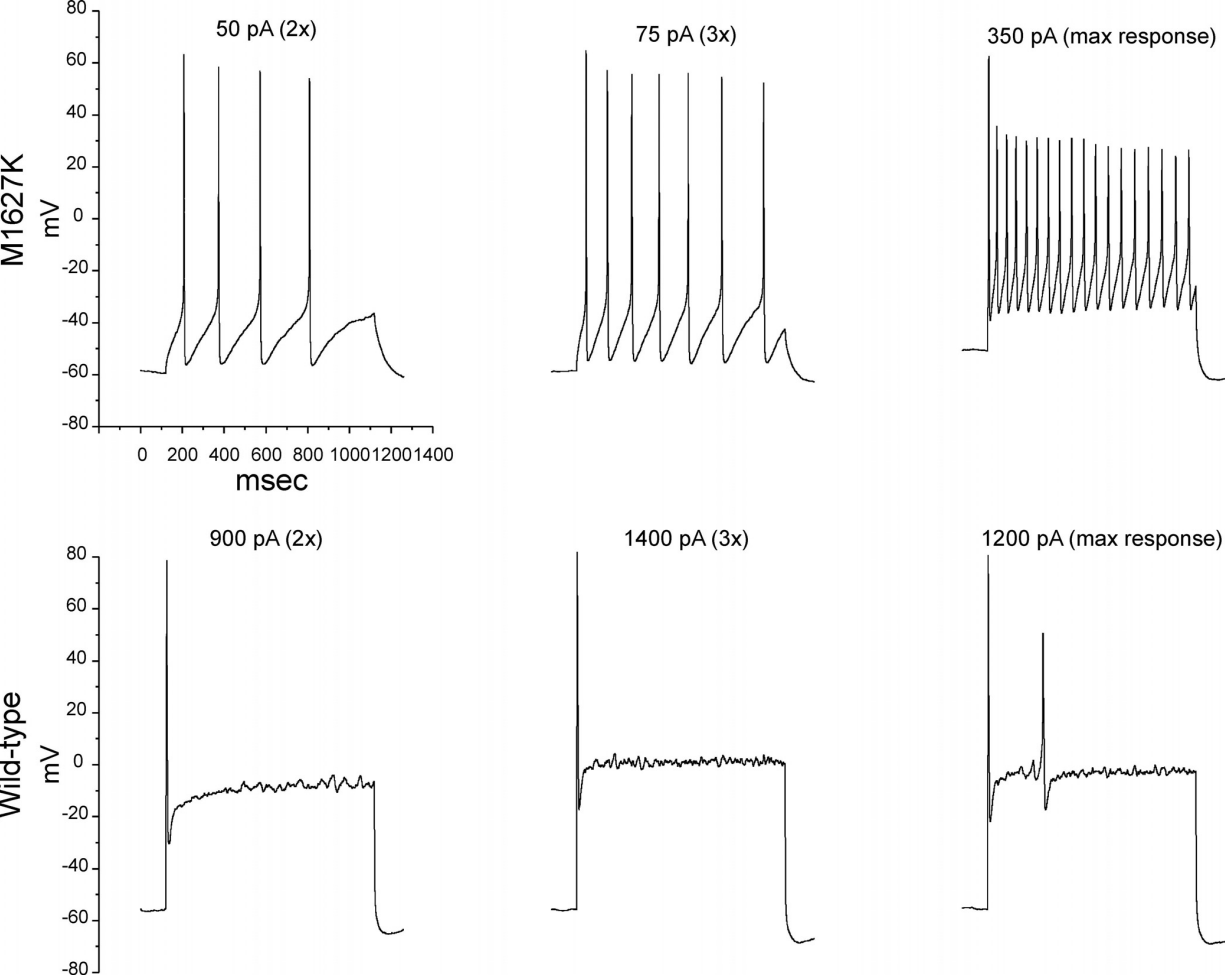
**Representative traces illustrating the differences in action potential firing in DRG neurons expressing either WT or M1627K channels**. The upper panels are the responses of a DRG neuron expressing M1627K channels in which the threshold for action potential generation was 25 pA. Panel A: stimulus intensity was 50 pA (2× threshold); panel B: stimulus intensity was 75 pA (3× threshold); panel C: stimulus was 350 pA (14× threshold) at which the peak rate was recorded. The lower panels are the responses of a DRG neuron expressing WT Nav1.7 channels in which the threshold for action potential generation was 450 pA. Panel D: stimulus intensity was 900 pA (2× threshold); panel E: stimulus was 1400 pA (3× threshold); panel F: shows that the peak response for this cell occurred at 1200 pA.

Higher rates of firing appeared to occur in M1627K cells with the lowest threshold values, which often did not reach their maximum firing rate until current injections of >10× threshold. In contrast, most of the WT transfected cells only produced a maximum of 1 or 2 action potentials even for current injections exceeding 1 nA (but still less than 4× threshold). Examples of these firing phenotypes are shown in Figure 9. The responses from a cell transfected with the M1627K mutant sodium channel in this case had a threshold of only 25 pA and reached a maximum response of 18 impulses/second to a current injection of only 350 pA. In contrast, this example of a WT transfected cell had threshold of 450 pA and fired a maximum of 2 spikes in response to a current injection of 1200 pA.

## Discussion

In this study, we identified a mutation, M1627K, in Na_v_1.7 from a previously unreported family with PEPD. Life-long symptoms in two members of the family are controlled by the sodium channel blocker carbamazepine. We investigated the functional effect of the M1627K substitution on Na_v_1.7, and confirm that it causes a large depolarizing shift in the voltage-dependence of steady-state fast-inactivation, with no effect on channel activation. Using current clamp, we show that M1627K mutant channels lower threshold for single action potentials and increase the number of action potentials in response to graded suprathreshold stimuli in small DRG neurons, most of which are nociceptors. Thus we show, for the first time, that a PEPD mutation produces nociceptor hyperexcitability.

Missense mutations in Na_v_1.7 have been linked to two types of inherited painful neuropathies, early-onset IEM [12-18], and PEPD [19]. While missense mutations in Na_v_1.7 have been found in most families with IEM and PEPD in whom the gene was sequenced, both IEM and PEPD may be genetically heterogenous because mutations in the coding exons of *SCN9A *were not found in 5 cases of PEPD [19], or in cases of familial early-onset [30] and adult-onset IEM [31], suggesting that other target genes or mutations in non-coding regions of *SCN9A *might underlie these cases. Mutations in the coding exons of sensory neuron-specific sodium channels Na_v_1.8 and Na_v_1.9 have been ruled out as causative in these cases of inherited erythromelalgia [30]. We present in this study a previously unreported familial case of PEPD from the UK with the mutation M1627K, in the DIV/S4–S5 linker, which is also present in a sporadic case of a male patient from France [19,20].

The patients in our study have responded favorably to treatment with carbamazepine, similar to those reported previously [20]. Carbamazepine targets voltage-gated sodium channels [32] and potently inhibits TTX-S channels in DRG neurons [33], including those specifically produced by wild-type Na_v_1.7 channels [34]. Although we did not examine the sensitivity of M1672K channels to carbamazepine in this study, carbamazepine has been shown to block persistent currents generated by I1461T and T1464I PEPD Na_v_1.7 mutant channels [19], and it is likely that inhibition of M1672K channel activity contributes to the therapeutic action of carbamazepine in the two patients examined in our study.

Our observations of a 19 mV depolarizing shift in the voltage-dependence of fast-inactivation for M1627K confirm the linkage of impaired fast-inactivation of Na_v_1.7 and PEPD reported by Fertleman et al [19]. We report here that recovery from fast-inactivation was accelerated in the M1627K, consistent with a destabilized inactivation state of the channel. We have also observed that M1627K display an increased ramp current, similar to IEM mutations [13,16,17,21-25]. M1627K displayed a trend toward a small (< 3 mV) hyperpolarizing shift in activation, but this was not statistically significant. Depolarizing shifts in fast-inactivation have also been observed in some [13,16,17] but not all [21-25] mutations that cause IEM. However, the depolarizing shifts in fast-inactivation associated to date with IEM (= 10 mV) are smaller than the shifts in PEPD mutations ([19] and this study). It is intriguing that the IEM mutation A863P with a +10 mV depolarizing shift in fast-inactivation [13,16,17] does not yield symptoms of PEPD, suggesting that other factors, perhaps genetic makeup or bigger shifts in the voltage-dependence of fast-inactivation may contribute to clinical manifestations of the disease.

Gating properties of Na_v_1.7, for example slow recovery from fast-inactivation and an ability to respond to ramp stimuli [28,35], suggest that, normally, it may act as a "threshold" channel which boosts subthreshold stimuli, and thus sets the gain in nociceptors [36,37]. Therefore, it is not surprising that mutations lowering the voltage-threshold for channel activation as in IEM lead to DRG neuron firing in response to a weaker stimulus that may normally be innocuous. Mutations that impair fast-inactivation as in PEPD allow more current to pass through the mutant channel, and thus induce stronger depolarization that brings the DRG neuron closer to the voltage-threshold for all-or-none action potential firing. By analogy to mutations in the cardiac channel Na_v_1.5 which cause arrhythmias and in neuronal channel Na_v_1.1 which causes epilepsy [26], the M1627K PEPD mutation which impairs fast-inactivation of Na_v_1.7 would be expected to increase repetitive firing, leading to hyperexcitablity of DRG neurons.

Indeed, we now show in this study that a PEPD mutation in human Na_v_1.7 channels in DRG neurons renders these cells hyperexcitable. Current clamp recordings showed a lower threshold for single action potentials, and an increased firing rate in response to suprathreshold stimuli, but did not show a change in resting membrane potential for DRG neurons expressing M1627K channels. However, Harty et al [13,16,17] have shown that depolarization of RMP produced by an IEM mutation (A863P) contributes to, but is not solely responsible for, the increase in DRG neuron hyperexcitability produced by that mutation. Thus, impaired fast-inactivation, accelerated repriming, and the enhanced response to slow depolarizations may all have contributed to the hyperexcitability of DRG neurons expressing M1627K.

The DIV/S4–5 linker is highly conserved in length and sequence (Figure 1C) among all sodium channels described to date, suggesting an important role in the normal functioning of the channel. Increasing the length of DIV/S4–S5 linker in Na_v_1.4 channels renders the mutant channels non-functional [38]. Importantly, mutations in this linker in several channels underlie pathological conditions [19,38-44]. Interestingly, substitution of the first methionine (M^a^) in this linker (Figure 1C) with a positively charged residue (M1627K) in Na_v_1.7 causes PEPD ([19] and this study), while substitution with a hydrophobic residue isoleucine (M1476I) in Na_v_1.4 causes cold-induced myotonia [42]. Subsitution of the second methionine (M^b^) with a positively charged residue (M1652R) in Na_v_1.5 causes LQT-3 syndrome [44]. All three disorders are linked to hyperexcitability of the cell in which they are expressed, irrespective if it is a neuron or a myocyte. The similarity of the outcome suggests a common mechanism of action, consistent with a conserved function of this linker in channel gating.

Site-directed mutagenesis studies have suggested that the DIV/S4–5 linker contributes to the receptor for the fast-inactivation tripeptide IFM in loop 3 (L3) which links DIII and DIV [45-47]. Structural studies have shown that this linker can acquire an α-helical structure [48] with several residues including the M^a^M^b ^(Figure 1C) forming a hydrophobic cluster that is important for inactivation, but indicate that these residues do not interact directly with the IFM motif [47,49,50]. Taken together, these studies suggest a model of two antiparallel α-helices [48]; this structure positions M^a^M^b ^to interact with Y^1470^Y^1471 ^(numbers according to Na_v_1.7) in L3. Substitution of the residues that correspond to M^a ^([19] and this study) or M^b ^[44] or Y^1470^Y^1471 ^residues destabilizes the inactivated state of the channel and yields similar gating changes in several channels. Interestingly, phosphorylation by Fyn kinase of Y^1495 ^which is predicted to interact with M^a ^in Na_v_1.5 (equivalent to Y^1471 ^in Na_v_1.7), produces a significant depolarizing shift in the voltage-dependence but no effect on the rate of steady-state fast-inactivation [51]. Thus the introduction of a charged residue at either of these two sites destabilizes this interaction and leads to impaired binding of the inactivation gate with its receptor.

In summary, our results show that a PEPD mutation produces hyperexcitability in DRG neurons. Our findings also confirm the impairment of fast-inactivation previously associated with PEPD mutations, but show that, in addition, a PEPD mutation can enhance the response of the Na_v_1.7 channel to small, slow depolarizations and accelerate repriming. These data contribute to a better understanding of the pathophysiology of pain in patients with PEPD and provide additional support for efforts to develop Na_v_1.7-specific therapeutics for treatment of neuropathic pain.

## Materials and methods

### Patients

The proband (Figure 1) is a 36 year old female with a history of erythema and burning pain in the lower parts of the body. Family consent was obtained according to an approved institutional review board protocol and blood samples were then withdrawn and analyzed for mutations in SCN9A.

### Exon screening

Genomic DNA (gDNA) was purified from venous blood. Human Caucasian variation panel DNA (25 males, 25 females; The Coriell Institute, Camden, NJ) was used as a normal population control. Coding exons and flanking intronic sequences were amplified and sequenced as described previously [13]. Briefly, PCR amplification was carried out using 150 ng gDNA, 1 μM primers and Expand Long Template polymerase (Roche, Indianapolis, IN) in a 50 μl reaction volume for 35 cycles (95°C × 30 s, 55°C × 30 s and 72°C × 1 min.). Short exons were amplified using two primers, whereas exon 26 required four sets of primers to cover its entirety. Genomic sequences were compared to the reference Na_v_1.7 cDNA [27] to identify sequence variation. Sequencing was performed at the Howard Hughes Medical Institute/Keck Biotechnology Center at Yale University. Sequence analysis used BLAST (National Library of Medicine) and Lasergene (DNAStar, Madison, WI).

### Voltage-clamp electrophysiology

The plasmid carrying the TTX-resistant (TTX-R) version of human Na_v_1.7 cDNA (hNa_v_1.7_R_) was previously described [35]. The M1627K mutation was introduced into hNa_v_1.7_R _using QuickChange XL II site-directed mutagenesis (Stratagene, La Jolla, CA). Transfected HEK 293 cells, grown under standard culture conditions (5% CO_2_, 37°C) in Dulbeccos's Modified Eagle's Medium supplemented with 10% fetal bovine serum, were treated with G418 for several weeks, and stable cell lines that express the mutant channel were selected.

Whole-cell patch-clamp recordings were conducted at room temperature (~21°C) using an EPC-10 amplifier and the Pulse program (v 8.5, HEKA Electronic, Germany). Fire-polished electrodes (0.8–1.5 MΩ) were fabricated from 1.7-mm VWR capillary glass using a Sutter P-97 puller (Novato, CA). Average access resistance was 2.1 ± 0.1 MΩ (mean ± SE, n = 27). Voltage errors were minimized using ~40–75% series resistance compensation to achieve identical (1.5 ± 0.2 mV) voltage error after series resistance compensation for the two groups. Capacitance artifacts were canceled using computer-controlled circuitry of the patch clamp amplifier and linear leak subtraction was used for all voltage clamp recordings. Recordings were always started 3.5–4 minutes after establishing the whole-cell configuration. Membrane currents were filtered at 5 kHz and sampled at 20 kHz. The pipette solution contained (in mM): 140 CsF, 1 EGTA, 10 NaCl and 10 HEPES (pH 7.3). The standard bathing solution was (in mM) 140 NaCl, 3 KCl, 1 MgCl2, 1 CaCl2, and 10 HEPES (pH 7.3). Data were analyzed using Pulsefit (HEKA Electronic, Germany) and Origin (Microcal Software, Northampton, MA) software. Unless otherwise noted, statistical significance was determined (p < 0.05) using an unpaired *t*-test. Results are presented as mean ± SEM and error bars in the figures represent standard errors (SE).

### Transfection of DRG neurons

The protocol for the care and sacrifice of rats used in the study was approved by the Veterans Administration CT Healthcare system IACUC. DRG tissue from 1- to 5-day old Sprague Dawley rats were harvested and dissociated using a protocol that was adapted from Rizzo et al [52]. Briefly, dissected ganglia were placed in ice cold oxygenated complete saline solution (CSS), which contained (in mM) 137 NaCl, 5.3 KCl, 1 MgCl_2_, 25 sorbitol, 3 CaCl_2_, 10 *N*-2-hydroxyethylpiperazine-*N' *-2-ethanesulfonic acid (HEPES); pH 7.2. They were then transferred to an oxygenated, 37°C CSS solution containing 1.5 mg/ml Collagenase A (Roche Applied Science, Indianapolis, IN) and 0.6 mM EDTA and incubated with gentle agitation at 37°C for 20 min. This solution was then exchanged with an oxygenated, 37°C CSS solution containing 1.5 mg/ml Collagenase D (Roche Applied Science, Indianapolis, IN), 0.6 mM EDTA and 30 U/ml papain (Worthington Biochemical, Lakewood, NJ) and incubated with gentle agitation at 37°C for 20 min. The solution was then aspirated and the ganglia triturated in DRG media [(DMEM/Fl2 (1:1) with 100 U/ml penicillin, 0.1 mg/ml streptomycin (Invitrogen, Carlsbad, CA) and 10% fetal calf serum (Hyclone, Logan, UT)], which contained 1.5 mg/ml bovine serum albumin (Sigma-Aldrich, St. Louis, MO) and 1.5 mg/ml trypsin inhibitor (Roche Applied Science, Indianapolis, IN).

Either WT hNa_v_1.7_R _or M1627K mutant channels were transiently transfected into the DRG neurons, along with enhanced-GFP, by electroporation with a Nucleofector II (Amaxa, Gaithersburg, MD) using Rat Neuron Nucleofector Solution and program G-013. The ratio of sodium channel to GFP constructs was 5:1. Immediately after transfection, the cells were allowed to recover for 5 minutes in Ca^2+^- and Mg^2+^-free culture medium (DMEM + 10% FBS). The cell suspension was then diluted with DRG media containing 1.5 mg/ml bovine serum albumin and 1.5 mg/ml trypsin inhibitor, 80 μl was plated on 12 mm circular poly-D-lysine/laminin pre-coated coverslips (BD Biosciences, Bedford, MA) and the cells incubated at 37°C in 5% CO_2 _for 30 min. DRG media (1 ml/well), supplemented with 50 ng/ml each of mNGF (Alomone Labs, Jerusalem, Israel) and GDNF (Peprotec, Rocky Hill, NJ), was then added and the cells maintained at 37°C in a 5% CO2 incubator for 18–48 hr before recording.

### Current-clamp electrophysiology

Small (20–30 μm diameter) GFP-labeled DRG neurons were used for current-clamp recording. Neurons with round cell body morphology and clear processes were selected for analysis and recordings were performed between 20- and 50-hours post-transfection. Pipette resistance was 1–3 MΩ when filled with the pipette solution which contained (in mM): 140 KCl, 0.5 EGTA, 5 HEPES, and 3 Mg-ATP, pH 7.3 with KOH (adjusted to 315 mOsm with dextrose). The extracellular solution contained the following (in mM): 140 NaCl, 3 KCl, 2 MgCl2, 2 CaCl2, 10 HEPES, pH 7.3 with NaOH (adjusted to 320 mOsm with dextrose). Formation of a GΩ seal and the transition to whole-cell configuration was performed in voltage-clamp mode before proceeding to the current-clamp recording mode. Recordings were obtained using an Axopatch 200B amplifier (Molecular Devices, Sunnyvale, CA) connected to a Digidata 1422 interface controlled by Clampex software (Molecular Devices). Cells with resting membrane potentials (RMP) more negative than -40 mV that were stable (<10% variation during the first 5 minutes) were included for data analysis. Input resistance was determined by fitting the slope of a line fit to hyperpolarizing voltage responses to current steps of -5 pA to -25 pA in 5 pA increments. Threshold was determined by the first action potential elicited by a series of depolarizing current injections that increased in 5 pA increments. Action potentials were counted by detecting membrane potential transients that exceeded the threshold value of 0 mV during 1-sec long depolarizing current injections. Data are expressed as means ± SEM. Student's t-test was used to assess the significance (p < 0.05) of differences between parameters measured from DRG neurons transfected with WT or M1627K mutant channels. Where indicated, the Mann-Whitney rank sum test was applied because of non-normal data distributions. Statistical analysis was performed using Sigmaplot software (Systat Software, San Jose, CA).

## Competing interests

The authors declare that they have no competing interests.

## Authors' contributions

SDD-H and TRC participated in the experimental design and interpretation of the data. ME and BJ collected, analyzed and interpreted electrophysiological data. LT identified the M1627K mutation, made the mutant M1627K construct and established the stable cell lines. TZF and ML collected and interpreted clinical data and confirmed a diagnosis of PEPD. SGW conceived the project, participated in the experimental design and interpretation. All authors participated in writing of the manuscript.
